# ﻿Comparative cytogenetics on eight Malagasy Mantellinae (Anura, Mantellidae) and a synthesis of the karyological data on the subfamily

**DOI:** 10.3897/compcytogen.v16.i1.76260

**Published:** 2022-02-11

**Authors:** Marcello Mezzasalma, Franco Andreone, Gaetano Odierna, Fabio Maria Guarino, Angelica Crottini

**Affiliations:** 1 CIBIO Research Centre in Biodiversity and Genetic Resources, InBIO, Universidade do Porto, Campus Agrário de Vairão, Rua Padre Armando Quintas, No 7, 4485-661 Vairão, Portugal; 2 BIOPOLIS Program in Genomics, Biodiversity and Land Planning, CIBIO, Campus de Vairão, 4485-661 Vairão, Portugal; 3 Museo Regionale di Scienze Naturali, Via G. Giolitti 36, 10123 Torino, Italy; 4 Department of Biology, University of Naples Federico II, Via Cinthia 26, 80126, Naples, Italy; 5 Departamento de Biologia, Faculdade de Ciências, Universidade do Porto, 4099-002 Porto, Portugal

**Keywords:** Amphibia, chromosome evolution, karyotype, Madagascar, NORs

## Abstract

We performed a molecular and cytogenetic analysis on different Mantellinae species and revised the available chromosomal data on this group to provide an updated assessment of its karyological diversity and evolution. Using a fragment of the mitochondrial 16S rRNA, we performed a molecular taxonomic identification of the samples that were used for cytogenetic analyses. A comparative cytogenetic analysis, with Giemsa’s staining, Ag-NOR staining and sequential C-banding + Giemsa + CMA + DAPI was performed on eight species: *Gephyromantis* sp. Ca19, *G.striatus* (Vences, Glaw, Andreone, Jesu et Schimmenti, 2002), Mantidactylus (Chonomantis) sp. Ca11, M. (Brygoomantis) alutus (Peracca, 1893), M. (Hylobatrachus) cowanii (Boulenger, 1882), Spinomantispropeaglavei “North” (Methuen et Hewitt, 1913), *S.phantasticus* (Glaw et Vences, 1997) and *S.* sp. Ca3. *Gephyromantisstriatus*, M. (Brygoomantis) alutus and Spinomantispropeaglavei “North” have a karyotype of 2n = 24 chromosomes while the other species show 2n = 26 chromosomes. Among the analysed species we detected differences in the number and position of telocentric elements, location of NOR loci (alternatively on the 6^th^, 7^th^ or 10^th^ pair) and in the distribution of heterochromatin, which shows species-specific patterns. Merging our data with those previously available, we propose a karyotype of 2n = 26 with all biarmed elements and loci of NORs on the 6^th^ chromosome pair as the ancestral state in the whole family Mantellidae. From this putative ancestral condition, a reduction of chromosome number through similar tandem fusions (from 2n = 26 to 2n = 24) occurred independently in Mantidactylus Boulenger, 1895 (subgenus Brygoomantis Dubois, 1992), *Spinomantis* Dubois, 1992 and *Gephyromantis* Methuen, 1920. Similarly, a relocation of NORs, from the putative primitive configuration on the 6^th^ chromosome, occurred independently in *Gephyromantis*, *Blommersia* Dubois, 1992, *Guibemantis* Dubois, 1992, *Mantella* Boulenger, 1882 and *Spinomantis*. Chromosome inversions of primitive biarmed elements likely generated a variable number of telocentric elements in *Mantellanigricans* Guibé, 1978 and a different number of taxa of *Gephyromantis* (subgenera *Duboimantis* Glaw et Vences, 2006 and *Laurentomantis* Dubois, 1980) and *Mantidactylus* (subgenera *Brygoomantis*, *Chonomantis* Glaw et Vences, 1994, *Hylobatrachus* Laurent, 1943 and *Ochthomantis* Glaw et Vences, 1994).

## ﻿Introduction

Madagascar is one of the richest biodiversity hotspots and an ideal region to study evolutionary dynamics (Myers et al. 2000; Ganzhorn et al. 2001; Vences et al. 2009). The native amphibians of Madagascar belong to four distinct anuran families: Hyperoliidae, Mantellidae, Microhylidae and Ptychadenidae (Glaw and Vences 2007). Among them, the family Mantellidae includes ca 230 described species (AmphibiaWeb 2021; Frost et al. 2021), representing the most species-rich amphibian group of the island.

Mantellidae are characterized by an extraordinary ecological and morphological diversity (Glaw and Vences 2007; Wollenberg et al. 2011; AmphibiaWeb 2021) and are subdivided into three subfamilies: Laliostominae with an overall low species diversity, including the genera *Laliostoma* Glaw, Vences et Böhme, 1998 (1 species) and *Aglyptodactylus* Boulenger, 1919 (6 species); Boophinae, a species-rich clade of about 80 described species of tree frogs, all belonging to the genus *Boophis* Tschudi, 1838, and Mantellinae, which is by far the most species-rich group including nine genera and more than 140 described species (Glaw and Vences 2007; AmphibiaWeb 2021).

The last three decades have seen the flourishing of the use of molecular techniques, with numerous taxonomic and systematic studies that clarified the relationships among the major groups within this subfamily (Glaw et al. 1998; Vences et al. 1998; Richards et al. 2000; Glaw and Vences 2006; Wollenberg et al. 2011; Kaffenberger et al. 2012). Similarly, these tools have been used in the identification of candidate species (Vieites et al. 2009; Perl et al. 2014) and have later contributed to the formal description of many of them (e.g. Andreone et al. 2003; Crottini et al. 2011a; Cocca et al. 2020; AmphibiaWeb 2021).

However, in contrast to the fast-growing amount of molecular data on Mantellidae, the available chromosomal data remain limited, leaving the karyological diversification of the family mostly unexplored. In particular, available cytogenetic data on the subfamily Mantellinae, obtained using different methods, come from a handful of studies (Morescalchi 1967; Blommers-Schlösser 1978; Pintak et al. 1998; Odierna et al. 2001; Andreone et al. 2003), together providing the description of the karyotype of ca. 40 species. These studies revealed the occurrence of a conserved chromosome number in most genera (2n = 26), but a marked difference in chromosome morphology, location of NORs and heterochromatin distribution (see Odierna et al. 2001 and Andreone et al. 2003). Differences in chromosome number (2n = 24) were also identified, with five species of the subgenus Brygoomantis all sharing this state, thus suggesting that the state of 2n = 24 is a derived feature of the group (Blommers-Schlösser 1978).

Comparative cytogenetics, especially when linked to phylogenetic inference, offers the possibility to identify plesio- and apomorphic states, and recognizes different evolutionary lineages (see e.g. Mezzasalma et al. 2013, 2014, 2017a). However, both the limited taxon sampling and the outdated taxonomy used in most previous works limited the possibility to draw robust comparisons and consistent hypotheses on the evolution of chromosomal diversification in the subfamily.

In this study we performed a comparative cytogenetic analysis on eight mantellid species belonging to the genera *Gephyromantis* Methuen, 1920, *Mantidactylus* Boulenger, 1895 (subgenera *Chonomantis*, *Brygoomantis* and *Hylobatrachus*) and *Spinomantis* Dubois, 1992, using a combination of standard coloration and banding methods. We coupled cytogenetic analyses with the molecular taxonomic identification of the samples and synthesized previously available information on this subfamily to produce an overview of their chromosomal diversity. This, enable us to propose a hypothesis on the chromosome diversification in mantelline frogs.

## ﻿Material and methods

### ﻿Sampling

We studied 13 samples of eight mantelline species belonging to the genera *Gephyromantis*, *Mantidactylus* (subgenera *Chonomantis*, *Brygoomantis* and *Hylobatrachus*) and *Spinomantis*. These samples were collected between 1999 and 2004 and conserved as cell suspensions at the University of Naples Federico II.

The list of samples used in this study is provided in Table 1. To provide an overview of the chromosomal data on Malagasy mantelline frogs, we reviewed previously published karyotypes of the subfamily. A complete list of all the considered taxa and karyotypes is provided in Table 2.

**Table 1.** Specimens analysed in this study. MRSN = Museo Regionale di Storia Naturale (Turin, Italy); ZMA = Zoological Museum Amsterdam (Amsterdam, Netherlands); FN and FAZC, field numbers of Franco Andreone; GA field numbers of Gennaro Aprea; FG/MV, field numbers of Frank Glaw and Miguel Vences.

Species	Field Number	Sex	Locality
*Gephyromantisstriatus*	MRSN A1988 (FN 7645)	female	Ambatoledama Corridor: Beanjada
*Gephyromantis* sp. Ca19	MRSN A2109 (FN 7630)	male	Ambatoledama Corridor: Beanjada
*Gephyromantis* sp. Ca19	MRSN A2075 (FN 7903)	male	Ambatoledama Corridor: Andasin’i Governera
*Gephyromantis* sp. Ca19	MRSN A2112 (FN 7890)	male	Ambatoledama Corridor: Andasin’i Governera
*Gephyromantis* sp. Ca19	MRSN A2108 (FN 7566)	female	Ambatoledama Corridor: Beanjada
Mantidactylus (Brygoomantis) alutus (Peracca, 1893)	MRSN A3639 (FN 7945)	female	Ankaratra: Manjakatompo
Mantidactylus (Chonomantis) sp. Ca11	MRSN A3708 (FN 7545)	male	Ambatoledama Corridor: Beanjada
Mantidactylus (Hylobatrachus) cowanii (Boulenger, 1882)	MRSN A2612 (FAZC 11370)	female	Antoetra: Soamazaka
Mantidactylus (Hylobatrachus) cowanii	GA 720	male	Mandraka
Spinomantispropeaglavei “North” (Methuen et Hewitt, 1913)	MRSN A3563 (FN 7543)	male	Ambatoledama Corridor: Beanjada
*Spinomantisphantasticus* (Glaw et Vences, 1997)	ZMA 19627 (FG/MV 2002-970)	male	Vohidrazana
*Spinomantis* sp. Ca3	MRSN A3998 (FN 7567)	male	Ambatoledama Corridor: Beanjada
*Spinomantis* sp. Ca3	MRSN A3999 (FN 7629)	male	Ambatoledama Corridor: Beanjada

**Table 2.** Available karyological data on mantelline frogs. M = metacentric pairs; sm = submetacentric pairs; st = subtelocentric pairs; t = telocentric pairs; AN = arm number; [#] = NOR bearing chromosome pair; CB = C-banding; F = Fluorochrome; R = references; (1) = Morescalchi (1967); (2) = Blommers-Schlösser (1978); (3) = Pintak et al. (1998); (4) = Odierna et al. (2001); (5) = Andreone et al. (2003); (6) = this study. Nomenclature follows Vieites et al. (2009), updated in Perl et al. (2014).

Genus/subgenus	Species	Karyotype	Banding	R
*Mantella* Boulenger, 1882	*aurantiaca* Mocquard, 1900	2n = 26 10m 3sm; AN = 52		(1)
	*aurantiaca*	2n = 26 10m 3sm; AN = 52		(2)
	*haraldmeieri* Busse, 1981	2n = 26 9m 4sm; AN = 52		(2)
	*ebenaui* (Boettger, 1880)	2n = 26 11m 2sm; AN = 52		(2)
	*aurantiaca*	2n = 26 11m 2sm; AN = 52	CB	(3)
	*crocea* Pintak et Böhme, 1990	2n = 26 11m 2sm; AN = 52	CB	(3)
	*baroni* Boulenger, 1888	2n = 26 11m 2sm; AN = 52	CB	(3)
	*haraldmeieri*	2n = 26 11m 2sm; AN = 52	CB	(3)
	*ebenaui*	2n = 26 11m 2sm; AN = 52	CB	(3)
	*viridis* Pintak et Böhme, 1988	2n = 26 11m 2sm; AN = 52	CB	(3)
	*laevigata* Methuen et Hewitt, 1913	2n = 26 11m 2sm; AN = 52	CB	(3)
	*baroni*	2n = 26 11m 2sm; AN = 52	Ag-NOR [2], CB	(4)
	*ebenaui*	2n = 26 11m 1sm; AN = 52	Ag-NOR [2], CB	(4)
	*betsileo* (Grandidier, 1872)	2n = 26 11m 2sm; AN = 52	Ag-NOR [2], CB	(4)
	*cowanii*	2n = 26 11m 2sm; AN = 52	Ag-NOR [2], CB	(4)
	*expectata* Busse et Böhme, 1992	2n = 26 11m 2sm; AN = 52	Ag-NOR [2], CB	(4)
	*laevigata*	2n = 26 11m 2sm; AN = 52	Ag-NOR [2], CB	(4)
	*madagascariensis* (Grandidier, 1872)	2n = 26 11m 1sm; 1st AN = 52	Ag-NOR [2], CB	(4)
	*nigricans* Guibé, 1978	2n = 26 10m 2sm; 1t AN = 48	Ag-NOR [2], CB	(4)
	*pulchra* Parker, 1925	2n = 26 11m 2sm; AN = 52	Ag-NOR [2], CB	(4)
	*viridis*	2n = 26 11m 2sm; AN = 52	Ag-NOR [2], CB	(4)
	*aurantiaca*	2n = 26 11m 2sm; AN = 52	Ag-NOR [2], CB	(4)
*Blommersia* Dubois, 1992	*blommersae* (Guibé 1975)	2n = 26 12m 1sm; AN = 52		(2)
	*galani* Vences, Köhler, Pabijan, et Glaw 2010	2n = 26 12m 1sm; AN = 52		(2)
	*grandisonae* (Guibé, 1974)	2n = 26 10m 3sm; AN = 52	Ag-NOR [1], CB, F	(5)
*Gephyromantis*				
*Asperomantis*	*asper* (Boulenger, 1882)	2n = 26 6m 3sm 4t; AN = 44		(2)
*Duboimantis*	*granulatus* (Boettger, 1881)	2n = 26 8m 4sm 1t; AN050	Ag-NOR [8], CB, F	(5)
*Duboimantis*	*leucomaculatus* (Guibé, 1975)	2n = 26 6m 6sm 1t; AN = 50	Ag-NOR [6], CB, F	(5)
*Duboimantis*	*luteus* (Methuen et Hewitt, 1913)	2n = 26 6m 4sm 1st 2t; AN = 48		(2)
*Duboimantis*	prope luteus Methuen et Hewitt, 1913	2n = 26 6m 2sm 1st 4t; AN = 42	Ag-NOR [11], CB, F	(5)
*Duboimantis*	propemoseri “Masoala” Glaw et Vences, 2002	2n = 26 6m 6sm 1t; AN = 52		(5)
*Duboimantis*	sp. Ca19	2n = 26 8m 5sm; AN = 52	Ag-NOR [6], CB, F	(6)
*Duboimantis*	*redimitus* (Boulenger, 1889)	2n = 26 7m 5sm 1t; AN = 50	Ag-NOR [6], CB, F	(5)
*Duboimantis*	*salegy* (Andreone, Aprea, Vences et Odierna, 2003)	2n = 26 5m 7sm 1st; AN = 52	Ag-NOR [6], CB, F	(5)
*Duboimantis*	*zavona* (Vences, Andreone, Glaw et Randrianirina, 2003)	2n = 26 9m 4sm; AN = 52	Ag-NOR [6], CB, F	(5)
*Laurentomantis*	*striatus*	2n = 24 6m 1sm 5t; AN = 38	Ag-NOR [10], CB, F	(6)
*Phylacomantis*	*pseudoasper* (Guibé, 1974)	2n = 26 7m 7sm; AN = 52	Ag-NOR [9], CB, F	(5)
*Guibemantis* Dubois, 1992				
*Guibemantis*	*depressiceps* (Boulenger, 1882)	2n = 26 10m 3sm; AN = 52		(2)
*Guibemantis*	*timidus* (Vences et Glaw, 2005)	2n = 26 11m 2sm; AN = 52		(2)
*Pandanusicola*	*methueni* (Angel, 1929)	2n = 26 11m 2sm; AN = 52		(2)
*Pandanusicola*	*bicalcaratus* (Boettger, 1913)	2n = 26 11m 2sm; AN = 52	Ag-NOR [1], CB, F	(4)
*Pandanusicola*	prope bicalcaratus (Boettger, 1913)	2n = 26 9m 4sm; AN = 52		(2)
*Pandanusicola*	*liber* (Peracca, 1893)	2n = 26 11m 2sm; AN = 52		(2)
*Pandanusicola*	*pulcher* (Boulenger, 1882)	2n = 26 9m 4sm; AN = 52		(2)
*Pandanusicola*	prope punctatus (Blommers-Schlösser, 1979)	2n = 26 10m 3sm; AN = 52	Ag-NOR [1], CB, F	(4)
*Pandanusicola*	*punctatus* (Blommers-Schlösser, 1979)	2n = 26 9m 4sm; AN = 52		(2)
*Mantidactylus*				
*Brygoomantis*	*alutus*	2n = 24 12m; AN = 48	Ag-NOR [6], CB, F	(6)
*Brygoomantis*	*ambohimitombi* Boulenger 1918	2n = 24 9m 3sm; AN = 48		(2)
*Brygoomantis*	*betsileanus* (Boulenger, 1882)	2n = 24 5m 6sm 1t; AN = 46		(2)
*Brygoomantis*	prope biporus (Boulenger, 1889	2n = 24 8m 4sm; AN = 48		(2)
*Brygoomantis*	sp. Ca19	2n = 24 7m 5sm; AN = 48		(2)
*Brygoomantis*	prope ulcerosus (Boettger, 1880)	2n = 24 8m 2sm 1st 1t; AN = 46		(2)
*Chonomantis*	prope aerumnalis (Peracca, 1893)	2n = 26 10m 2sm 1t; AN = 50		(2)
*Chonomantis*	sp. Ca11	2n = 26 10m 2sm 2t; AN = 50		(6)
*Chonomantis*	*paidroa* Bora, Ramilijaona, Raminosoa et Vences, 2011	2n = 26 6m 7sm; AN = 52		(2)
*Hylobatrachus*	*cowanii* (Boulenger, 1882)	2n = 26 12m 1t; AN = 50	Ag-NOR [6], CB, F	(6)
*Hylobatrachus*	*lugubris* (Duméril, 1853)	2n = 26 9m 3sm 1t; AN = 50		(2)
*Mantidactylus*	*guttulatus* (Boulenger, 1881)	2n = 26 11m 2sm; AN = 52		(2)
*Ochthomantis*	propefemoralis (Boulenger, 1882)	2n = 26 9m 3sm 1t; AN = 50		(2)
*Spinomantis*				(2)
	*aglavei* (Methuen et Hewitt, 1913)	2n = 24 9m 3sm; AN = 48		(2)
	prope aglavei “North”	2n = 24 10m 2sm; AN = 48	Ag-NOR [7], CB, F	(6)
	*peraccae* (Boulenger, 1896)	2n = 26 7m 6sm; AN = 48		(2)
	*phantasticus*	2n = 26 13m; AN = 52		(6)
	sp. Ca3	2n = 26 12m 1sm: AN = 52	Ag-NOR [6], CB, F	(6)

### ﻿Molecular taxonomic identification

DNA was extracted from cell suspensions following Sambrook (1989). A 3’ fragment of ca. 550 bp of the mitochondrial 16S rRNA gene was amplified using the primer pair 16Sa (CGCCTGTTTATCAAAAACAT) and 16Sb (CCGGTCTGAAACTCAGATCAGT) (Palumbi et al. 1991). This marker proved to be suitable for amphibian identification (Vences et al. 2005) and has been widely used for Malagasy amphibians (e.g. Vieites et al. 2009; Rosa et al. 2012; Crottini et al. 2011b, 2014; Penny et al. 2017). Amplification conditions were: initial denaturation at 94 °C for 5 min, 36 cycles at 94 °C for 30 s, 50 °C for 45s and 72 °C for 45 s, followed by a final step at 72 °C for 7 min. Amplicons were sequenced on an automated sequencer ABI 377 (Applied Biosystems, Foster City, CA, USA) using BigDye Terminator 3.1 (ABI). Chromatograms were checked and edited using Chromas Lite 2.6.6 and BioEdit 7.2.6.1 (Hall 1999). All newly determined sequences were deposited in GenBank (accession numbers: OL830846–OL830858). For taxonomic attribution we compared newly generated sequences with a curated database of reference sequences of the 3’ terminus of the 16S gene for all lineages of Malagasy mantellid frogs (Cocca 2020). Taxonomic attribution was performed using a local BLAST analysis against this reference database.

### ﻿Chromosomal analysis

Cell suspensions were obtained from tissue samples as described in Mezzasalma et al. (2013). In brief, tissues were incubated for 30 min in hypotonic solution (KCl 0.075 M + sodium citrate 0.5%, 1:1) and fixed for 15 min in methanol-acetic acid, 3:1. Fixed tissues were stored at 4 °C and dissociated manually on a steel sieve. Chromosomes were obtained using the air-drying method and stained with conventional colorations (5% Giemsa solution at pH 7), Ag-NOR staining (Howell and Black 1980), C-banding according to Sumner (1972) and sequential C-banding + Fluorochromes (CMA+DAPI) following Mezzasalma et al. (2015). Ag-NOR and C-banding staining were not performed on *M.* sp. Ca11 and *S.phantasticus*, because quantity and quality of metaphase plates were not adequate for additional staining methods. Karyotype reconstruction was performed using at least five plates per sample.

## ﻿Results

### ﻿Molecular taxonomic identification

The selected 16S fragment was successfully amplified and sequenced from all analysed samples. All newly generated sequences showed identity scores > 97% with homologous sequences available in the mantellid frogs database generated in Cocca (2020). We followed the nomenclature used in Vieites et al. (2009), updated in Perl et al. (2014) (see Table 1).

### ﻿Cytogenetic analysis

**Figure 1.**
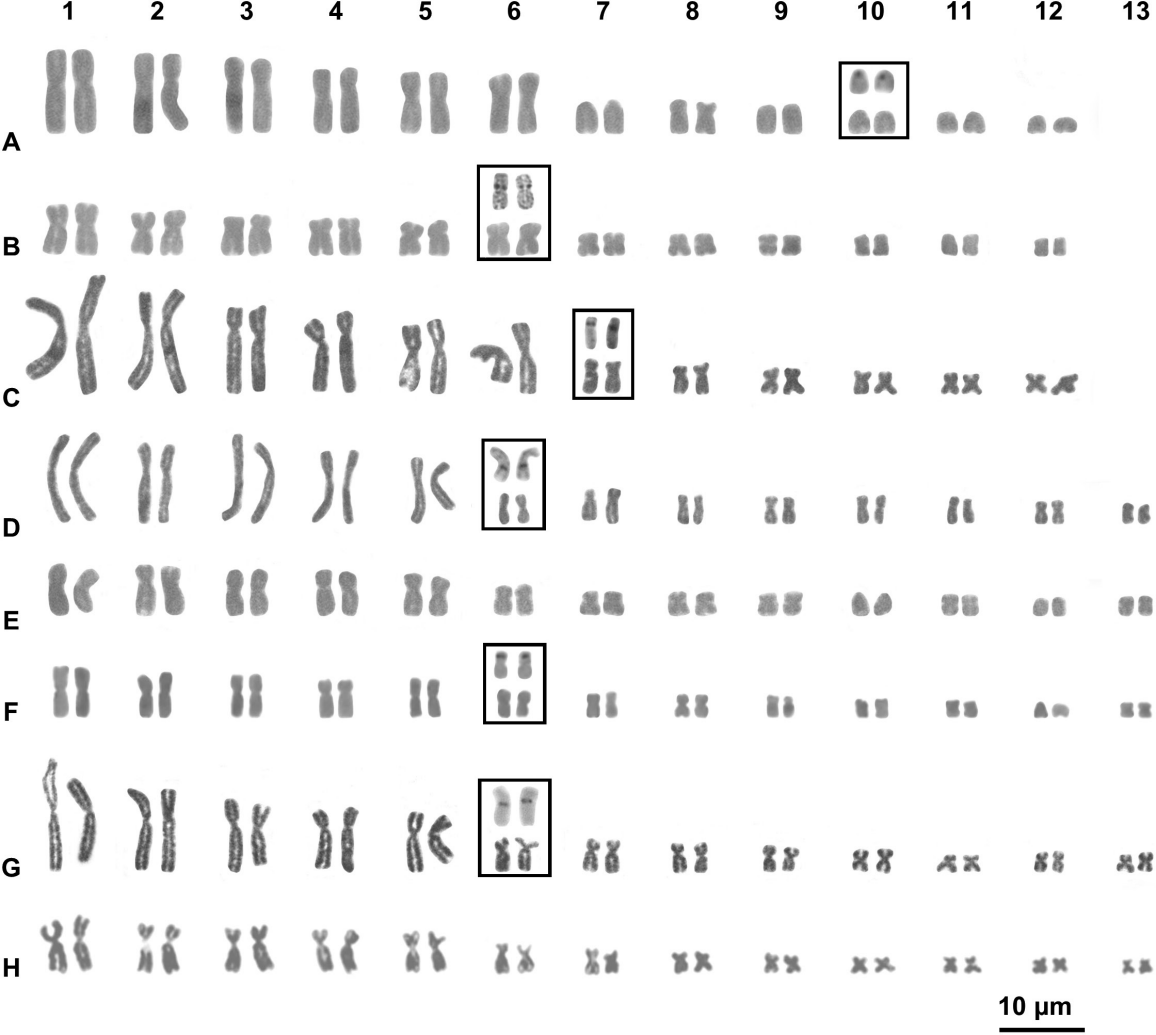
Giemsa stained karyotypes of **A***Gephyromantisstriatus* (FN 7645) **B**Mantidactylus (Brygoomantis) alutus (FN 7945) **C**Spimomantispropeaglavei “North” (FN 7543) **D***Gephyromantis* sp. Ca19 (FN 7630) **E**Mantidactylus (Chonomantis) sp. Ca11 (FN 7545) **F**Mantidactylus (Hylobatrachus) cowanii (FAZC 11370) **G***Spinomantis* sp. Ca3 (FN 7567) and **H***Spinomantisphantasticus* (FG/MV 2002-970). Insets represent NOR-bearing pairs stained with Giemsa (down in the insets) and Ag-NOR method (up in the insets).

The studied specimen of *Gephyromantisstriatus*, Mantidactylus (Brygoomantis) alutus and Spinomantispropeaglavei “North” have a karyotype of 2n = 24 chromosomes, with the first six pairs distinctively larger than the other six pairs (Fig. 1; Table 3). In *G.striatus*, the pairs 1–6, 8 are biarmed while the other pairs are telocentric, with the pair 10 bearing the NOR loci (Fig. 1A; Table 3). In M. (Brygoomantis) alutus and S.propeaglavei “North” all pairs are biarmed and NOR loci were detected on the 6^th^ and 7^th^ pair (Fig. 1B, C), respectively.

**Table 3.** Chromosome morphometric parameters of the study species. LR%= % Relative Length (length of a chromosome/total chromosome length*100); CI = centromeric index (ratio between short arm/chromosome length*100). Sh = chromosome shape (m = metacentric; sm = submetacentric; t = telocentric).

Sp.	*G.striatus*	*M.alutus*	S.propeaglavei	*G.* sp. Ca19	*M.* sp Ca11	*M.cowanii*	*S.* sp. Ca11	*S.phantasticus*
Chr.	LR%-CI	LR%-CI	LR%-CI	LR%-CI	LR%-CI	LR%-CI	LR%-CI	LR%-CI
	(sh)	(sh)	(sh)	(sh)	(sh)	(sh)	(sh)	(sh)
**1**	16.8–41.6	15.1–44.0	16.9–40.7	15.0–46.3	12.3–39.3	18.6–48.8	16.1–37.8	16.2–38.5
	(m)	(m)	(m)	(m)	(m)	(m)	(m)	(m)
**2**	12.7–36.9	11.8–48.5	14.0–32.0	13.7–35.6	12.0–34.9	12.9–42.3	14.2–42.8	13.8–30.9
	(m)	(m)	(sm)	(sm)	(sm)	(m)	(m)	(sm)
**3**	11.8–36.7	11.6–34.1	12.1–26.0	12.4–40.8	11.2–43.9	12.8–37.2	12.4–38.2	11.5–34.8
	(sm)	(sm)	(sm)	(m)	(m)	(sm)	(m)	(sm)
**4**	10.9–39.0	10.6–41.1	11.9–34.3	11.3–42.8	11.1–38.4	11.3–40.0	12.1–30.6	11.4–38.5
	(m)	(m)	(sm)	(m)	(m)	(m)	(sm)	(m)
**5**	10.2–45.2	10.2–44.6	9.7–44.7	10.6–36.1	10.0–41.7	19.2–44.8	9.1–36.0	10.4–35.1
	(m)	(m)	(m)	(sm)	(m)	(m)	(sm)	(sm)
**6**	9.7–48.7	10.1–48.2	9.7–42.6	6.4–31.1	6.2–44.7	5.3–47.3	5.5–38.2	6.2–33.2
	(m)	(m)	(m)	(sm)	(m)	(m)	(m)	(sm)
**7**	6.0–0	5.9–49.0	4.5–33.0	5.0–40.1	6.1–46.2	5.3–49.3	5.5–38.7	6.2–42.9
	(t)	(m)	(m)	(m)	(m)	(m)	(m)	(m)
**8**	5.6–39.0	5.9–41.4	4.1–47.0	4.8–29.3	6.1–41.0	4.8–49.6	5.1–39.8	5.9–44.5
	(m)	(m)	(m)	(sm)	(m)	(m)	(m)	(m)
**9**	5.4–0	5.8–45.8	3.9–47.0	4.4–48.8	5.9–43.8	4.4–34.4	4.9–43.9	4.4–48.8
	(t)	(m)	(m)	(m)	(m)	(sm)	(m)	(m)
**10**	4.6–0	4.9–43.0	3.5–39.3	4.3–42.9	5.5–0	4.3–41.7	4.2–44.1	3.8–48.8
	(t)	(m)	(m)	(m)	(t)	(m)	(m)	(m)
**11**	3.4–0	4.1–45.0	3.3–49.0	4.3–37.4	5.5–47.5	4.2–40.8	3.6–41.7	3.7–44.1
	(t)	(m)	(m)	(sm)	(m)	(m)	(m)	(m)
**12**	2.9–0	4.0–46.3	3.1–47.4	4.2–37.4	4.2–0	4.0–0	3.4–38.0	3.5–49.6
	(t)	(m)	(m)	(sm)	(t)	(t)	(m)	(m)
**13**				3.6–43.5	4.1–42.6	3.8–38.2	3.2–46.1	3.0–43.8
				(m)	(m)	(m)	(m)	(m)

The samples of the other five species (*G.* sp. Ca19, M. (Chonomantis) sp. Ca11, M. (Hylobatrachus) cowanii, *S.phantasticus* and *S.* sp. Ca3) presented a karyotype of 2n = 26 chromosomes, with the first five pairs distinctively larger than the remaining eight pairs (Fig. 1D–H). In these species, all chromosome pairs resulted biarmed, with the exception of *M.cowanii* and of M. (Chonomantis) sp. Ca11, whose karyotype showed one (pair 12) and two pairs (10 and 12) composed of telocentric elements, respectively (Fig. 1E, F). The sixth pair is the NOR bearing one in *G.* sp. Ca19, *M.cowanii* and *S.* sp. Ca3 (Fig. 1D, F, G).

**Figure 2.**
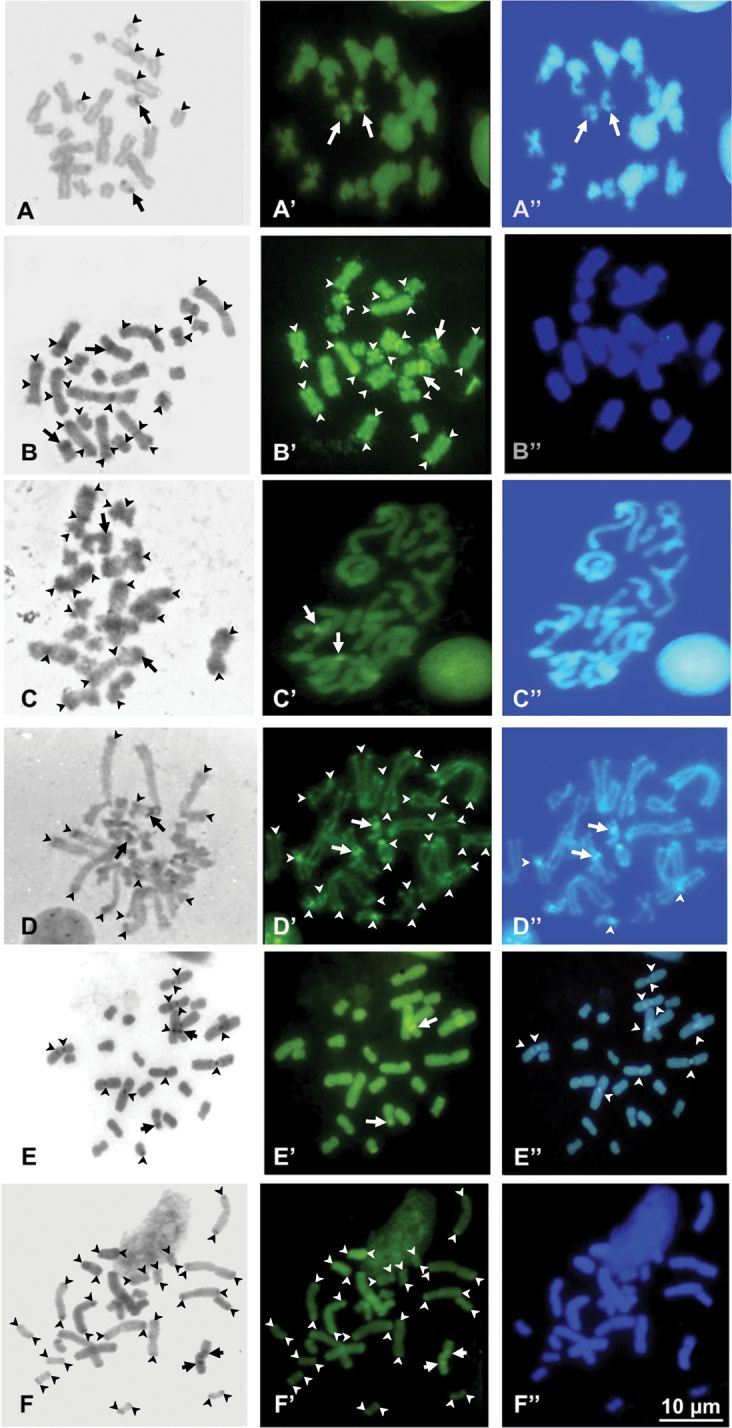
Metaphase plates of *Gephyromantisstriatus* (**A, A’, A**”), Mantidactylus (Brygoomantis) alutus (**B, B’, B**”), Spinomantispropeaglavei “North” (**C, C’, C**”), *Gephyromantis* sp. Ca19 (**D, D’, D**”), *Spinomantis* sp. Ca3 (**E, E’, E**”) and Mantidactylus (Hylobatrachus) cowanii (**F, F’, F**”) stained with C-banding + Giemsa (**A–F**), + CMA (**A’–F**’) + DAPI (**A”–F**”). Arrows point at NORs while arrowheads highlight other heterochromatin blocks.

In *G.striatus*, NOR associated heterochromatin was C-banding positive (CMA + and DAPI -) and tiny centromeric C-bands were present on some chromosome pairs (Fig. 2A, A’, A”). *Mantidactylusalutus* and Spimomantispropeaglavei “North” showed centromeric and telomeric C-bands and NOR associated heterochromatin which were positive to CMA and DAPI negative (Fig. 2B, B’, B” and C, C’, C”). Mantidactylus (Brygoomantis) alutus also presented an additional bright centromeric band on the chromosomes of pair nine. *Gephyromantis* sp. Ca19 showed centromeric and telomeric C- bands, which were CMA and DAPI positive (Fig. 2D, D’, D”). *Spinomantis* sp. Ca3 showed solid telomeric C-bands and NOR associated heterochromatin, which resulted CMA positive and DAPI negative (Fig. 2E, E’, E”). Mantidactylus (Hylobatrachus) cowanii had centromeric C-bands on all chromosomes, which were CMA and DAPI negative (Fig. 2F, F’, F”). No heteromorphic or completely heterochromatic chromosome were found in any of the studied samples.

## ﻿Discussion

We here provide new karyological data on eight frog species belonging to the subfamily Mantellinae and discuss the available chromosome data on this subfamily to provide a first comprehensive assessment of its karyological diversity.

Available data on representatives of the other two Mantellidae subfamilies (Boophininae and Laliostominae) highlight the occurrence of a conserved karyotype structure in terms of chromosome number and morphology. In particular, the first karyological studies by Blommers-Schlössers (1978) on 12 species of *Boophis* (Boophininae) and on *Aglyptodactylusmadagascariensis* (Duméril, 1853) (Laliostominae) revealed a conserved karyotype of 2n = 26 with all biarmed chromosomes.

Following studies by Aprea et al. (1998, 2004) expanded the knowledge on the karyological uniformity to the position of NORs loci, invariably on the sixth chromosome pair both in *Boophis* and *A.madagascariensis*, but evidenced different patterns of heterochromatin composition and distribution. Similar karyological characters were described also in different species of the genus *Mantella* (belonging to the subfamily Mantellinae), all showing a karyotype of 2n = 26 with all biarmed chromosomes (Blommers-Schlössers 1978; Odierna et al. 2001). A karyotype of 2n = 26 with all biarmed elements should thus be considered the primitive condition in the whole family Mantellidae, as it is highly conserved in all subfamilies, genera and most subgenera (see Blommers-Schlösser 1978; Aprea et al. 1998, 2004; Odierna et al. 2001, see Table 2). Nevertheless, species of other genera of the subfamily Mantellinae show a wider karyological variability, both concerning chromosomes number, morphology, localizations of NORs loci and heterochromatin composition and distribution (Blommers-Schlösser 1978; Odierna et al. 2001; present study) (see also Table 2).

**Figure 3.**
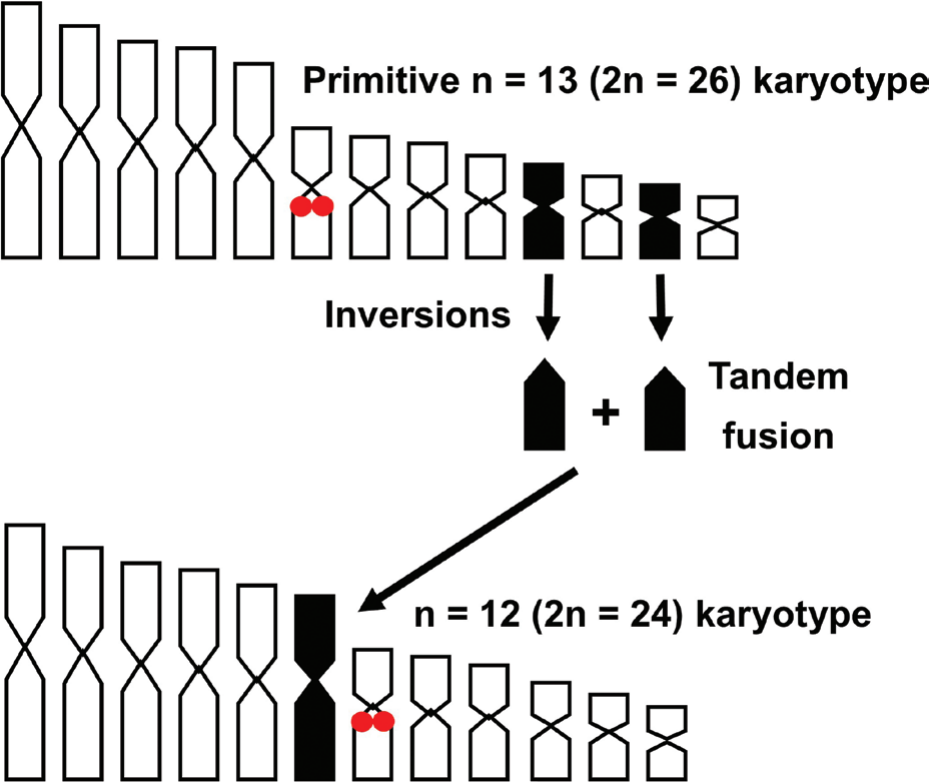
Hypothesized general model of chromosome reduction in Mantellinae from n = 13 (2n = 26) to n = 12 (2n = 24) by means of chromosome fusions. Red dots highlight the NOR bearing chromosome.

Concerning the variability of the chromosome number, a 2n = 26 karyotype is still the most common chromosomal configuration, but karyotypes with a reduced chromosome complement (2n = 24) have been documented in 9 species of three different genera (6 species of Mantidactylus (subgenus Brygoomantis), 2 *Spinomantis* and *Gephyromantisstriatus*) (See Fig. 1 and Table 2). Furthermore, while the 2n = 26 configuration occurs in all three subfamilies of the family Mantellidae (Mantellinae, Boophinae and Laliostominae) (e.g. Aprea et al. 1998, 2004; present study), karyotypes with 2n = 24 seem to occur in just a few phylogenetically lineages (genus *Gephyromantis*, *Mantidactylus* and *Spinomantis*), where the 2n = 26 configuration is also present (Blommers-Schlösser 1978; present study). In turn, the subfamily Boophinae, with all the species showing a 2n = 26 karyotype (Aprea et al. 1998, 2004), has been depicted as a basal group in the Mantellidae radiation (see e.g. Wollenberg et al. 2011). These evidences suggest that a reduction of the chromosome number from 2n = 26 to 2n = 24 occurred repeatedly and independently in different lineages of the subfamily Mantellinae, probably involving chromosome inversions and a fusion (translocation) between two elements of the smallest pairs (6–13), giving rise to an additional large (6^th^) chromosome pair in several species (e.g. *G.striatus*, M. (Brygoomantis) alutus, and S.propeaglavei “North”) (Fig. 3; Table 3). Interestingly, a similar reduction of the chromosome number driven by tandem fusions (from 2n = 26 to 24) has been documented also in the family Ranidae (Miura et al. 1995).

Other than tandem fusions, chromosome inversions of primitive biarmed elements also had a significant role in the morphological chromosome diversity observed in mantelline frogs. These mechanisms generated a variable number of telocentric elements in different evolutionary lineages (see Figs 1, 3 and Table 2).

Considering the position of the loci of NORs, our results and available literature data (Aprea et al. 1998, 2004; Odierna et al. 2001; Andreone et al. 2003), show that NORs occurrence on the sixth chromosome pair can be considered a primitive state, as it is described for all analysed species belonging to the genus *Boophis*, *A.madascariensis* and most *Gephyromantis*, Mantidactylus (subgenus Brygoomantis), and *Spinomantis*. On the other hand, a derivate configuration of NOR loci seems to have emerged multiple times in distinct lineages. The different positions of NOR loci in mantelline frogs suggest that these elements were also differently involved in the hypothesized chromosome fusions from 2n = 26 to 2n = 24, providing further support to multiple, independent rearrangements leading to similar karyotype configurations. In fact, while in M. (Brygoomantis) alutus the sixth large chromosome pair likely derived from a fusion involving the primitive NOR bearing pair and another smaller pair, in *G.striatus* and S.propeaglavei “North” the pair 6 does not include NOR loci, which are found on the 7^th^ and 10^th^ chromosome pair, respectively (see Fig. 1). In other species of *Gephyromantis*, *Blommersia*, *Guibemantis* and *Mantella* the relocation of NORs involved different pairs (1^st^, 2^nd^, 8^th^, 9^th^, 10^th^ or 11^th^) (Odierna et al. 2001; Andreone et al. 2003; this study). It should be noted that Ag-NOR staining only evidences active NORs, and the existence of different inactive sites in the karyotypes of the studied species cannot be excluded based only on this analysis. However, we found correspondence in NOR location using both Ag-NOR and C-banding + CMA (in Figs 1, 2), which also has the power to uncover rDNA clusters (Schmid 1982; Zaleśna et al. 2017).

Various mechanisms may be responsible for NOR relocation, such as cryptic structural rearrangements, minute insertions, reintegration of rDNA genes amplified during ovogonial auxocytosis or the activation of silent sites (Nardi et al. 1977; Schmid 1978; King 1980; Mahony and Robinson 1986; Schmid and Guttenbach 1988; Mezzasalma et al. 2018). These mechanisms may be independent to other rearrangements, despite the resulting change in the configuration of NORs is a significant indicator of lineage divergence at different taxonomic level (e.g. Pardo et al. 2001; Mezzasalma et al. 2015, 2018, 2021).

Sequential C-banding did not evidence the occurrence of any sex-specific, largely heterochromatic chromosomes (generally related to differentiated heterogametic sex chromosomes, a condition not yet documented in the family Mantellidae), B chromosomes, or interchromosomal rearrangements leading to heteromorphic autosome pairs (e.g. Mezzasalma et al. 2014, 2016, 2017b; Sidhom et al. 2020). Nevertheless, C-banding showed a heterogeneous heterochromatin distribution in Mantellidae (see also Aprea et al. 1998, 2004; Odierna et al. 2001; Andreone et al. 2003), highlighting the occurrence of species-specific banding patterns. For example, *G.striatus* and M. (Hylobatrachus) cowanii show different amount and location of C-banding positive heterochromatin in comparison with closely related species with the same chromosome number and similar morphology (e.g. *G.* sp. Ca19 and M. (Brygoomantis) alutus). Interspecific variations in heterochromatin are generally due to different levels of amplification of highly repetitive DNA (Charlesworth et al. 1994). These differences mostly occurred without modifications of the chromosome morphology in Mantellidae (see also Aprea et al. 1998, 2004; Odierna et al. 2001; Andreone et al. 2003), probably by means of symmetrical addition/deletion of heterochromatin. The occurrence of distinctive species-specific banding patterns may be useful in evolutionary cytogenetic and cytotaxonomic studies in the subfamily, but comprehensive comparative analyses would benefit from more banding data on species of different genera and subgenera.

Finally, we also highlight the importance of a preliminary molecular taxonomic identification of mantellid frogs for a consistent karyotype attribution, and that future cytogenetic studies should focus on *Laliostoma*Glaw et al., 1998, *Wakea* Glaw et Vences, 2006, *Boehmantis* Glaw et Vences, 2006 and *Tsingymantis* Glaw et al., 2006, as well as on different undersampled genera and subgenera.

## ﻿Conclusions

We provide new chromosomal data on eight species belonging to the subfamily Mantellinae, advancing the knowledge on their karyotype diversity, and suggesting that a reduction in the chromosome number and the relocation of NORs loci occurred repeatedly and independently in different genera of this subfamily. We hypothesize a karyotype of 2n = 26 with all biarmed elements and loci of NORs on the 6^th^ chromosome pair as the ancestral state in the whole family Mantellidae and propose a model for the reduction of the chromosome number from 2n = 26 to 2n = 24 by means of tandem fusions.
